# Improvement of RBD‐FC Immunogenicity by Using Alum–Sodium Alginate Adjuvant Against SARS‐COV‐2

**DOI:** 10.1111/irv.70018

**Published:** 2024-10-30

**Authors:** Mahboobeh Dehghan, Hossein Askari, Masoud Tohidfar, Seyed Omid Ranaei Siadat, Fataneh Fatemi

**Affiliations:** ^1^ Department of Cellular and Molecular Biology, Faculty of Life Sciences and Biotechnology Shahid Beheshti University Tehran Iran; ^2^ Protein Research Center Shahid Beheshti University Tehran Iran

**Keywords:** aluminum hydroxide, cytokine, immunogenicity, RBD‐FC, SARS‐CoV‐2, sodium alginate, vaccine

## Abstract

**Background:**

Adjuvants use several mechanisms to boost immunogenicity and to modulate immune response. The strength of adsorption of antigen by adjuvants can be a determinant factor for significant improvement of immunopotentiation.

**Methods:**

We expressed recombinant RBD‐FC in PichiaPink Strain 4 and examined the vaccination of mice by vaccine formulation with different adjuvants (sodium alginate and aluminum hydroxide, alone and together).

**Results:**

Sodium alginate significantly increased the immunogenicity and stability of RBD‐FC antigen, so RBD‐FC formulated with combined alginate and alum (AlSa) and sodium alginate alone showed higher antibody titer and stability. Immunogenicity of RBD‐FC:AlSa was determined by serological assays including direct enzyme‐linked immunosorbent assay (ELISA) and surrogate virus neutralization test (sVNT). High levels of IgGs and neutralizing antibodies were measured in serum of mice immunized with the RBD‐FC:AlSa formulation. On the other hand, cytokines IL‐10 and INF‐γ were severely accumulated in response to RBD‐FC:AlSa, and after 10 days, their accumulation was significantly declined, whereas IL‐4 showed the highest and the lowest accumulation in response to alum and alginate, respectively.

**Conclusions:**

Our data may suggest that combination of alum and sodium alginate has a better compatibility with RBD‐FC in vaccine formulation.

## 1 Introduction

SARS‐COV‐2 is a new member of COVs that include a large group of diverse enveloped single‐stranded RNA viruses [1]. Coronaviruses have four open reading frames (ORFs) that encode structural proteins [2]. Spike protein of coronavirus has two major functional subunits (N‐terminal S1 and C‐terminal S2), of which residues 319–591 from S1 correspond to the receptor‐binding domain (RBD) and are responsible for interaction with angiotensin‐converting enzyme 2 (ACE2) [3]. The RBD undergoes essential conformational changes to introduce viral material into the cell [2, 4]. Previous studies demonstrated that SARS‐CoV RBD contains multiple conformation‐dependent epitopes that are able to induce high‐titer neutralizing antibodies [5, 6]. It is suggested that RBD is one of the most important targets for developing SARS vaccine [7]. Antibodies against RBD with humoral immune response seem to prevent the binding of RBD to ACE2 and consequently inhibit entry of the virus into the host cell [8]. On the other hand, it has been shown that the fc fragment of human IgG in the RBD‐based vaccine, RBD‐Fc, can act as a remarkable immune enhancer to increase the immunogenicity of RBD [6, 7, 9].

According to the efforts being made to produce an efficient vaccine, it is necessary to use adjuvants that meet safety and efficacy criteria. There is a general tendency to use endogenous and exogenous natural adjuvants as a potential source of immunomodulatory compounds [10, 11] such as *Leishmania infantum* eukaryotic initiation factor [12], Toll‐like‐receptor agonists [13], triterpenoid saponins [14], hyaluronic acid [15], liposomes [16], some emulsions [17], and sodium alginate [18]. Aluminum hydroxide (Alum) is currently the most common adjuvant in the vaccines, though it creates obstacles such as weak induction of immune response and cold‐sensitive suspension that need to be overcome [19]. Manufacturing of aluminum hydroxide with the same physiochemical properties, like other mineral adjuvants, is difficult, and this incompatibility negatively affects the immunogenicity of alum‐based formulation. In addition, vaccines containing alum cannot be sterilized by standard methods and are not able to tolerate freezing and freeze‐drying procedures [20].

Sodium alginate is a natural and biodegradable polysaccharide of sodium alginic acid molecules used as a superior adjuvant [21] for viral vaccines [22], which shows that some alginate derivatives can effectively inhibit the viral polymerase activity [23] and inactivate enveloped viruses like SARS‐CoV‐2 [24]. Since 1950, sodium alginate was introduced as a vaccine adjuvant [25, 26], and after that until 1965, Flowers [27], Jaros and Dewey [28], and, finally, Scherr et al. [21] reported sodium alginate with various formulations as new adjuvant. Until now, sodium alginate was extensively used as an efficient adjuvant for different vaccine formulations. Alginate‐based biomaterials are capable of inhibiting a wide variety of viruses (wide range of 17 types of viruses) that can infect different organisms like humans, mice, plants, and bacteria [29]. Sodium alginate in combination with recombinant RBD increased immunogenicity of RBD antigen. Interestingly, the combination of alum and sodium alginate significantly increases some cytokine levels than sodium alginate alone [30].

In this study, a methylotrophic yeast, *Pichia pastoris*, was used as a host organism for the expression of spike glycoprotein RBD. We have designed a recombinant antigen, RBD‐Fc, which contains RBD of S1 protein of SARS‐CoV‐2 and Fc fragment of human IgG to assess Fc fragment functionality to boost immunogenicity and vaccine efficacy in combination with different formulation of RBD‐FC antigen with two kind of adjuvants, sodium alginate and alum on mice by competitive ELISA and sVNT. Also, this study provides evidence to show adjuvant of alum‐alginate stably induced inflammatory cytokine levels in mice sera immunized with RBD‐Fc.

## 2 Materials and Methods

### 2.1 Chemical Materials

All reagents were purchased from Merck unless stated otherwise. The RBD gene was synthesized by Biomatik USA LLC. Plasmid extraction kit and gel recovery kit were prepared from Sangon Biotech (Shanghai) Co. Ltd. (China). All cloning materials were purchased from Thermo Fisher Scientific (GeneArt, Regensburg, Germany). The protein A resin, goat anti‐mouse IgG antibody (HRP conjugated), and goat anti‐human IgG antibody (HRP conjugated) were obtained from Sigma‐Aldrich (USA). SARS CoV‐2 neutralizing Ab ELISA kit, PichiaPink Strain 4, Ppink‐α‐HC vector, ELISA kit for cytokine assay, and PAD selection plates were purchased from Invitrogen, USA. Balb‐C mice were purchased from Razi Vaccine & Serum Research Institute (IRAN). Animals have been housed in accordance with WHO guidelines and standard laboratory conditions (WHO guidelines on nonclinical evaluation of vaccines of 2005). Animal research were approved by Iran National Committee by Ethics in Biomedical Research.

### 2.2 Transformation, Production, and Purification of Recombinant RBD‐Fc Protein

The RBD gene (residues V320–G550) of SARS‐CoV‐2 strain Wuhan‐Hu‐1 (GenBank ID: MN908947.3) was codon‐optimized and synthesized. The optimized RBD gene bearing an Fc tag at c terminal region was inserted into the backbone of vector Ppink‐α‐HC [31], yielding plasmid Ppink‐α‐HC‐RBD as described by Zang et al. [32]. In brief, the plasmid (Ppink‐α‐HC‐RBD) was linearized with BSPE1 and transformed into competent PichiaPink Strain 4 by electroporation. After transformation, the yeast cells were plated onto Pichia Adenine Dropout (PAD) selection plates containing ampicillin and lacking adenine. After 48‐h incubation time, 10 colonies were randomly picked, and colony polymerase chain reaction (PCR) was performed. Each colony was suspended in 50 μL of sterile water, and 10 μL of that was used as DNA template. The reaction conditions were 95°C for 10 min, followed by 30 cycles of denaturation at 95°C for 30 s, annealing at 58°C for 45 s and extension 72°C for 80 s. The last step of the reaction was a final elongation at 72°C for 10 min and stored at 4°C.

To mass production of the RBD protein, four selected yeast clones were separately cultured in 4000 mL yeast extract–peptone–glycerol (YPG) medium, pH 6 at 28°C with continuous shaking until optical density was reached to 0.8–0.9. The yeast cells were pelleted by centrifugation and then resuspended in 4000 mL yeast extract–peptone (YP) medium, pH 7.5 with 1% methanol. After 48–72‐h induction at 28°C, the culture supernatants were harvested and concentrated with ammonium sulfate 80%. The presence of RBD‐Fc protein was verified on 12% SDS‐PAGE, followed by silver staining. RBD‐Fc proteins were then purified using protein A resin according to manufacturer's instruction protocol.

### 2.3 Western Blot Analysis

The purified recombinant RBD protein was separated by 12% SDS‐PAGE and transferred onto polyvinylidene difluoride (PVDF) membrane. After blocking by tris‐buffered saline (TBS) 1× (pH 7.5) containing 5% skim milk for 1 h at room temperature, the membrane was washed and then incubated with anti‐human IgG‐HRP (horseradish peroxidase) conjugated antibodies (1:2000 dilution) for 1 h at room temperature. After washing with TBST (TBS + Tween 20) buffer for 5 min, 3,3′‐diaminobenzidine tetrahydrochloride (DAB) was applied to visualize the reaction [32].

### 2.4 Vaccine Formulation and Injection

Sodium alginate (Sa) and aluminum hydroxide gel (Al) adjuvants were prepared with two concentrations of 5 and 0.5 mg/mL, respectively [19]. Different adjuvants were used to obtain a final concentration of 100 μg/mL of RBD antigen (Table 1). The different vaccines, Al (100 μg/mL RBD antigen and 0.5 mg/mL alum), Sa (100 μg/mL RBD antigen and 5 mg/mL sodium alginate), AlSa (100 μg/mL RBD antigen, 0.5 mg/mL alum, and 5 mg/mL sodium alginate), and normal saline as control treatment were prepared and incubated for 2 h at room temperature on a shaker to complete absorption of the antigen and adjuvant. Combination of Al with Sa (AlSa) was prepared by adding Al to the antigen and then incubated for 1 h on a shaker at room temperature, and finally, Sa was added and incubated again for 1 h at the same condition.

**TABLE 1:** Vaccine components and formulations.

Vaccine components	Concentration	Unit	Vaccine formulations (μL)			
			Control	Al	Sa	AlSa
Normal saline	0.9	%	100	85	65	60
RBD antigen	100	μg/mL	—	10	10	10
Alum	0.5	mg/mL	—	5	—	5
Sodium alginate	5	mg/mL	—	—	25	25

Twenty‐four BALB/c female mice aged 4–6 weeks were randomly divided into four groups and subcutaneously vaccinated in the dorsum of the neck with 100 μL/mouse [33]. The second injection of the vaccine was carried out after 21 days from the first injection with the same protocol. Blood samples were collected at 10 days after each vaccine injection in order to measure IgG concentration.

### 2.5 Evaluation of the Loading Efficacy of the Vaccine Formulations

The loading efficiency of each vaccine formulation was indirectly measured by quantifying the free antigen (RBD) remaining in the supernatant after centrifugation of the vaccine mixture at 10,000 rpm for 10 min. The concentration of antigen in the supernatant was determined using the BSA standard curve by the Lowry method, and the loading efficacy (LE) was calculated based on the following equation [34].

$$\begin{array}{c} \mathrm{LE}\left(\%\right)=(\left(\text{Total amount of}\;\mathrm{RBD}-\text{Free}\;\mathrm{RBD}\;\text{amount}\right)\\ /\left(\text{Total amount of}\;\mathrm{RBD}\right))\times 100 \end{array}$$

### 2.6 Determination of RBD‐Fc‐Specific Antibodies Titer by ELISA

The serum of the blood samples was collected by centrifugation for 10 min at 14000 g. As described by Darvish et al. [30], for titration of RBD‐Fc‐specific antibodies in mouse antisera, purified RBD (1 μg/mL) was resuspended in 50 mM carbonate buffer (pH 9.6), and 100 μL of it was distributed inside the ELISA plate. After incubating the coated plate at 4°C overnight, it was incubated with blocking buffer, phosphate‐buffered saline (PBS) including 2% bovine serum albumin (BSA) for 2 h at 37°C. The plate was washed four times with PBST (PBS + 0.05% Tween 20), and then 100 μL of each serum samples, which were twofold serially diluted in blocking solution starting from a 1:40 dilution, was added to the wells. After incubating the plate for 90 min at 37°C, HRP‐conjugated goat anti‐mouse IgG antibody was added and incubated one more hour at 37°C. 3,3′,5,5′‐Tetramethylbenzidine(TMB) was added (100 μL/well) as a substrate after washing 4 times with PBST. The reaction was stopped by adding 50 μL of 0.2 M sulfuric acid, and the absorbance was read after 10 min at 450/630 nm in an ELISA device (Biotech, USA).

### 2.7 Inhibition ELISA

Immunogenetic specificity of serum antibody against RBD antigen and its inhibition rate were determined by competitive or inhibitory ELISA method [35]. In brief, different amounts of antigen (concentration ranging from 1 to 40 μg/mL) were incubated with mice sera groups after immunization with different vaccine formulations for 2 h at 37°C, and it was incubated again at a temperature of 4°C for an overnight. Then, 100 μL of the formed immune complex (antigen serum) was added to the wells covered by RBD (we1ls coated with 1 μg/mL RBD) instead of the mice sera. The rest of the steps were performed like indirect binding ELISA. Also, as a control, each sera group was incubated with assay buffer instead of antigen. As expected, the control wells “No Antigen Control” (NAC) showed the maximum OD. By using the following equation, the inhibition percentage of different wells containing samples (antigen serum) against NAC wells was calculated, where A_inhibited_ and A_NAC_ are the absorbance of inhibited and no antigen control wells, respectively.

$$\text{Inhibition}\%=\left(A_{\mathrm{NAC}}-A_{\text{inhibited}}\right)/A_{\mathrm{NAC}}\times 100$$

### 2.8 SARS‐CoV‐2 Surrogate Virus Neutralization Test (sVNT)

Surrogate virus neutralization test (sVNT) was assayed according to SARS‐CoV‐2 neutralizing antibody test kit by ELISA method [33]. In this kit, the plate wells are coated with RBD antigen (1 μg RBD per 100 μL buffer per each well), and the series of standards are evaluated with concentrations (0, 1, 2.5, 5, 10, and 40 μg/mL) of SARS‐COV‐2 neutralizing antibody with confirmed SARS‐COV‐2 neutralization performance. Fifty microliters of each standard and samples are poured into the respective well in such a way that the first six wells were used for different standards and the next wells were used for positive and negative controls and the other wells were used for samples. Immediately, 50 μL of HRP conjugated hACE2 was added to the corresponding wells. The contents of the wells were mixed slowly, and the wells were incubated for 30 min at 37°C. After 30‐min incubation and 5 times washing with PBST, 100 μL of TMB as substrate was added to each well. The wells were placed in the dark for 15 min at room temperature. By adding 100 μL of stop solution to each well, the optical absorption of each well was measured using an ELISA reader at 450 and 630 nm.

### 2.9 Cytokine Measurement

The concentration of IFN‐γ, IL‐10 and IL‐4 as key cytokines were measured in the collected sera from each group of mice at 10 and 20 days after second vaccine injection. The measurement was carried out by using Invitrogen ELISA kit with the basis of manufacture instruction protocol.

### 2.10 Statistical Analysis

All measured parameters were tested using the two‐way ANOVA by using SPSS software (Version 13.0). Each treatments had three replications, and significant differences among treatments were determined by Fisher's least significant difference test (LSD) with *p* ≤ 0.05. With the basis of LSD test, means with the same letter are not significantly different from each other, and error bars showed ±standard deviation of the means.

## 3 Results

### 3.1 Production of Recombinant Monomeric RBD of SARS‐CoV‐2 in Yeast

**FIGURE 1:**
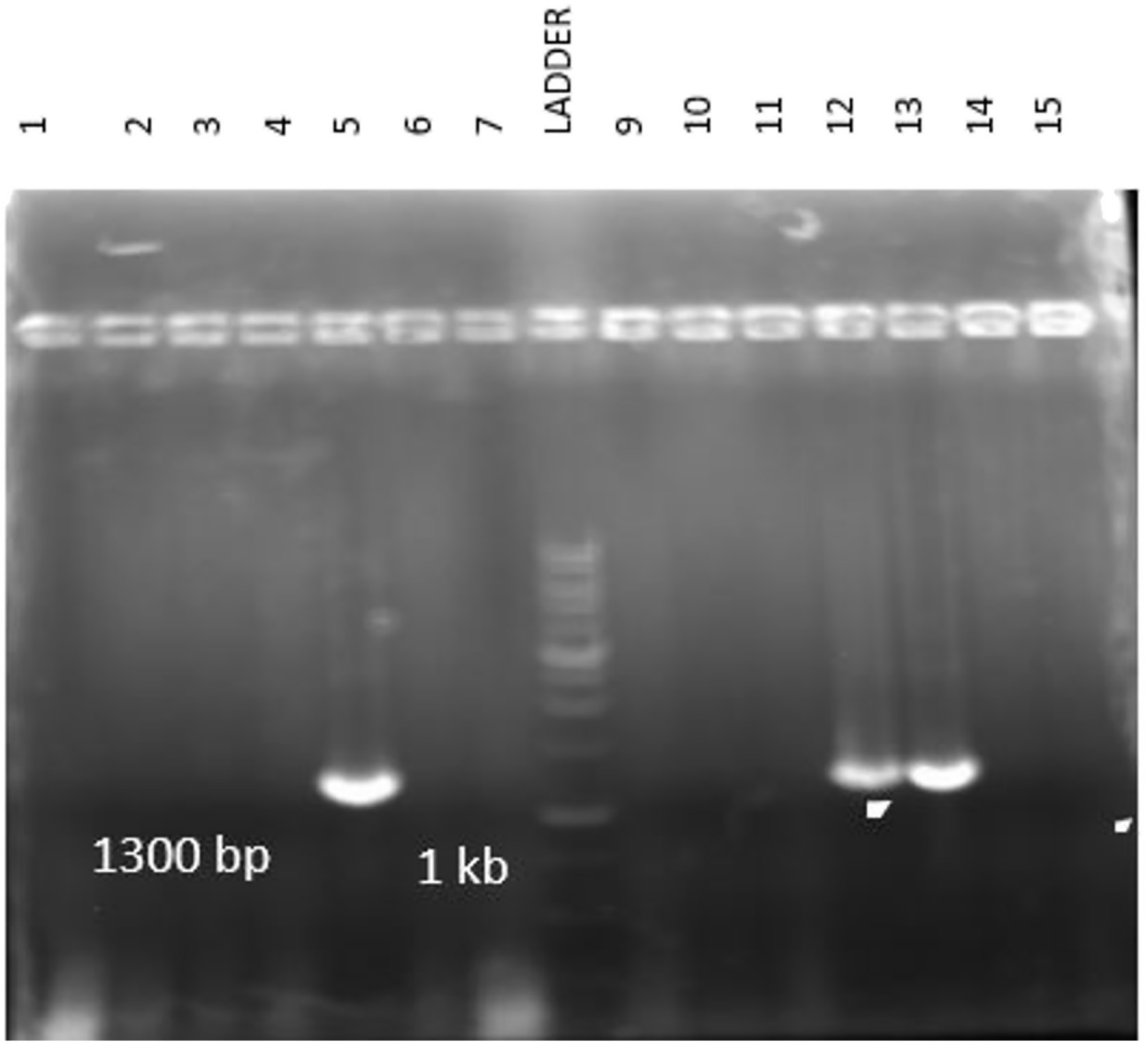
Several transformed yeast colonies were selected on nutrient medium and assessed for successful transformation by using colony PCR. DNA marker and the 1300 bp RBD‐Fc bands in Lanes 5, 12, and 13 have been shown.

**FIGURE 2:**
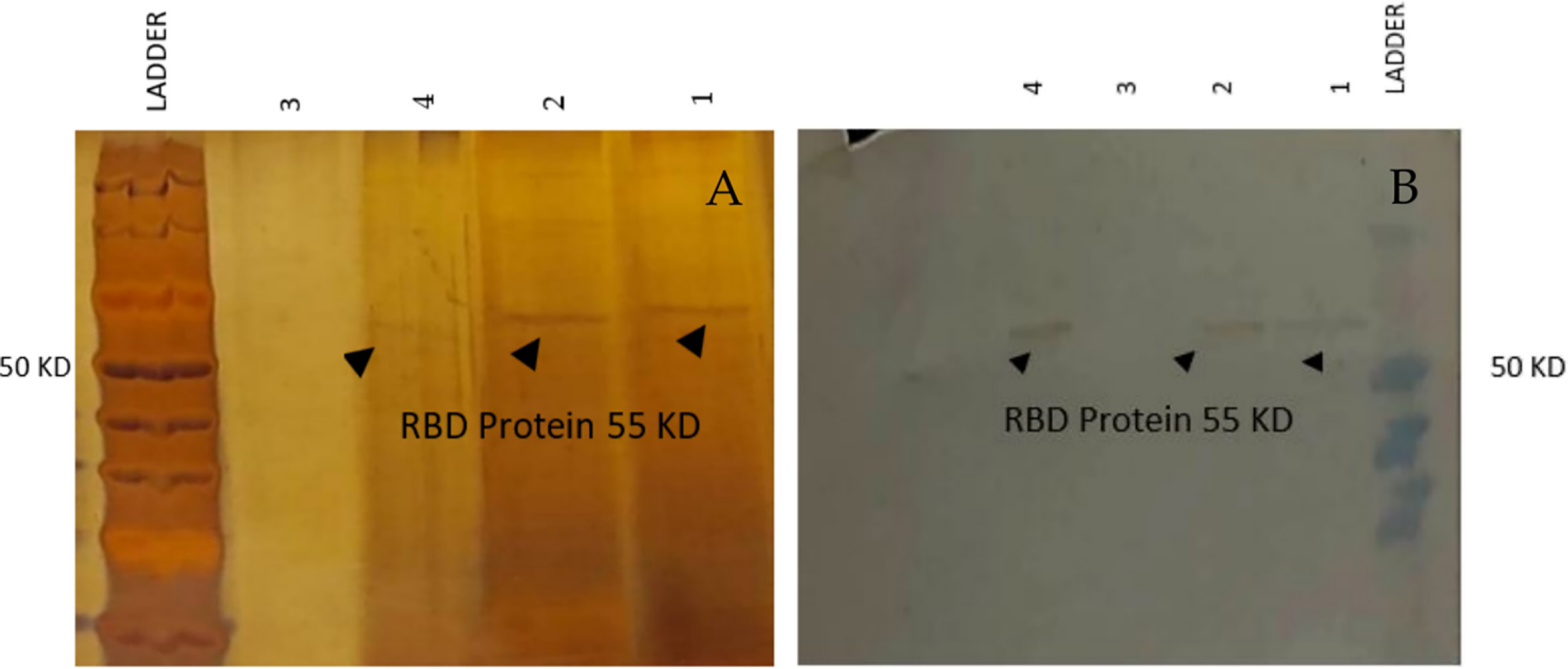
(A) Verification of RBD‐Fc presence in the yeast culture supernatants by analytical SDS‐PAGE 12%. Four samples were assessed and of them samples 1, 2, and 4 had detectable RBD‐Fc. (B) Western blot assay of RBD protein. Purified RBD was run on SDS‐PAGE 12% with prestained protein marker in lane ladder. The gel was transferred on PVDF membrane and probed by mouse monoclonal anti‐human IgG Fc (HRP) (Lanes 1, 2, and 4). Control (−) pPinkα‐HC induced for 96 h (Lane 3).

To produce SARS‐CoV‐2 RBD recombinant protein in yeast, an expression vector termed pPinkα‐HC‐RBD was constructed. This vector encoded SARS‐CoV‐2 RBD (residues 319–591) fused with an N‐terminal α‐mating factor signal peptide and a C‐terminal Fc‐tag. The pPinkα‐HC‐RBD vector was used to transform *P. pastoris* yeast. The yeast transformants were analyzed by using colony PCR to confirm the presence of RBD fraction (Figure 1), and finally, the transformants with positive PCR results were chosen for RBD expression analysis. Out of 10 randomly selected colonies, three colonies successfully transformed and produced RBD‐Fc protein (Figures 1 and 2). To mass production of RBD by the selected yeasts, YP medium treated with 1% methanol was employed, and then RBD‐FC protein was extracted, concentrated, and purified, and finally, our western blot analysis by using anti‐human IgG‐HRP conjugated antibody showed that all three colonies are good primary candidates for mass production of RBD‐Fc protein (Figure 2).

### 3.2 Loading Efficacy Test

**FIGURE 3:**
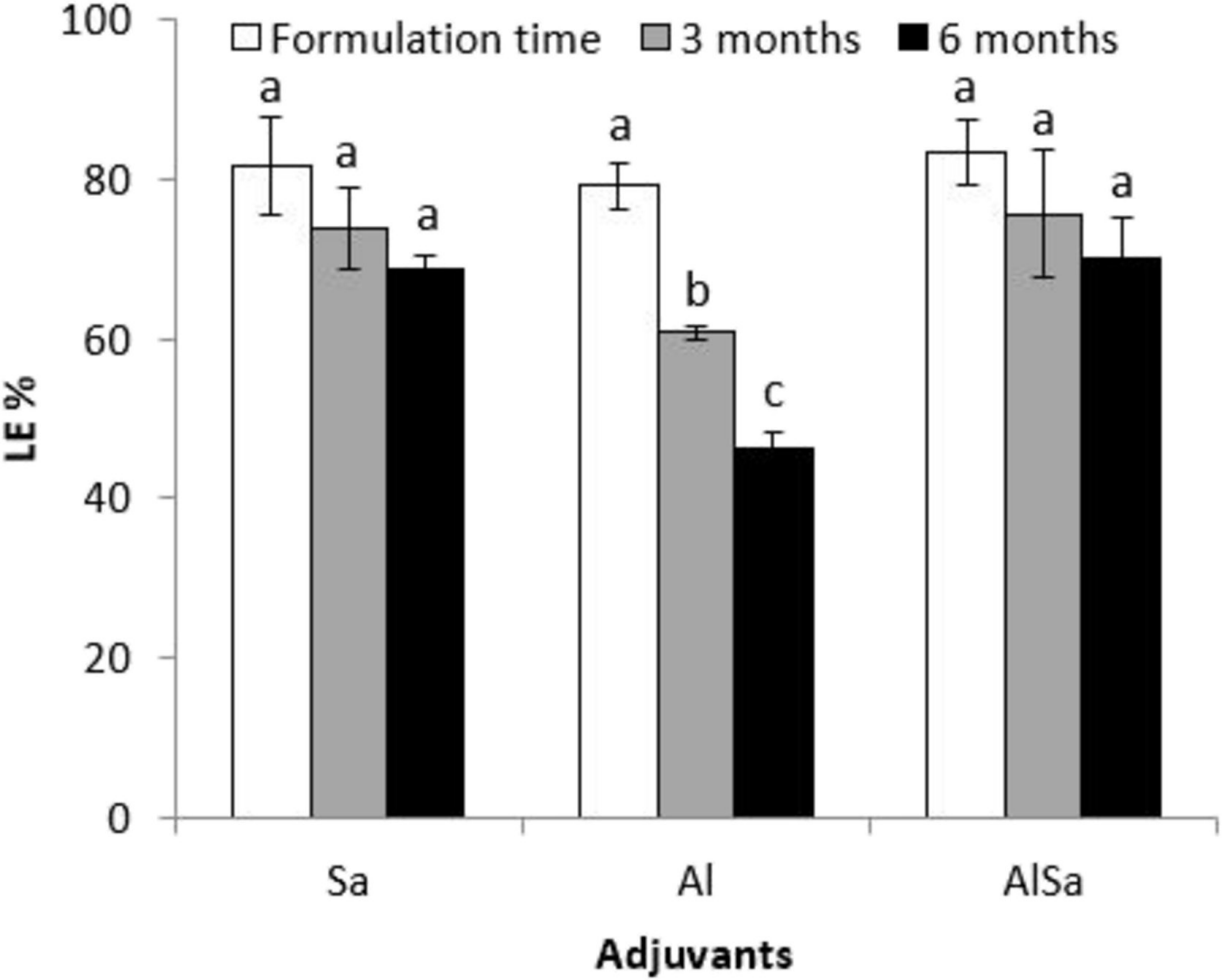
Loading efficacy assessment during three time points showed that alum–sodium alginate combination and sodium alginate have the highest stability of loading efficacy in comparison with alum adjuvant.

One of the major challenges in vaccine formulation is time dependency of loading efficacy. Here, we examine the effect of two time points on the loading efficacy of antigen adsorption to the adjuvants. The presented results showed that the lowest loading efficacy belongs to alum adjuvant, whereas the other formulations containing sodium alginate (Sa and AlSa) significantly demonstrated higher stable loading efficacy (Figure 3). During the experiment, decrease in loading efficacy in formulation containing sodium alginate was up to 9%, whereas in vaccine formulation containing only alum, the decrease amount was about 25%, in average. The data may be suggested that sodium alginate acts as a major player for stable loading efficacy.

### 3.3 Elicitation of Protective Neutralizing Antibodies in Mice

**FIGURE 4:**
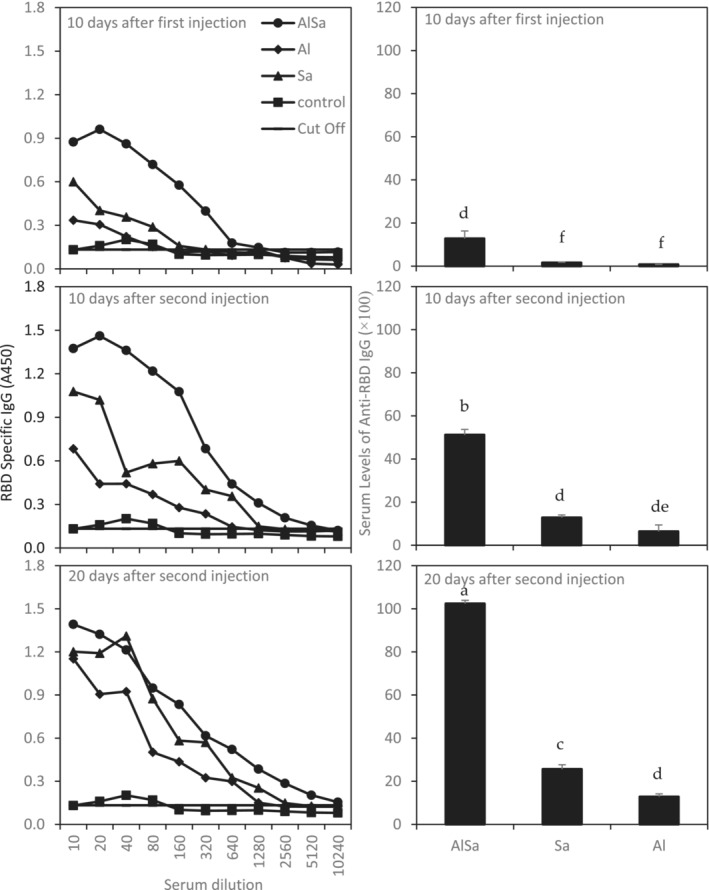
Mice immunized with RBD‐Fc produced RBD‐specific IgG. Left figures show RBD‐specific IgG in different dilutions of sera from 10 days after first, 10 days after second, and 20 days after second injections using ELISA assay. Right figures show serum levels of anti‐RBD IgG from 10 days after first, 10 days after second, and 20 days after second infections using ELISA assay. As presented, IgG level increased along with all kinds of adjuvants, and 20 days after booster (second) injection, the highest level of anti‐RBD IgG has been achieved. Besides, AlSa was an effective player in generation of anti‐RBD IgG.

Yeast‐derived monomeric RBD successfully elicited anti‐RBD IgG in comparison with control group (Figure 4). To achieve this result, mice were immunized with three kinds of candidate vaccines in two doses. Blood samples were collected 10 days after first and second vaccination and 20 days after boost and analyzed for the anti‐RBD IgG. None of the serum samples from the control group that were treated with NaCl 0.9% exhibited any significant binding activity even at the lowest serum dilution tested (1:10). In contrast, serum samples from RBD‐immunized mice exhibited significant binding activity after the first blood draw, and the RBD‐specific antibody titers increased greatly at second blood draw. As presented in Figure 4, adjuvant AlSa reveals a severe effectiveness in elicitation of anti‐RBD‐IgG at all three sampling times, whereas sodium alginate had an effective impact only on Day 20 after boost than alum adjuvant (Figure 4).

### 3.4 Inhibition Test

**FIGURE 5:**
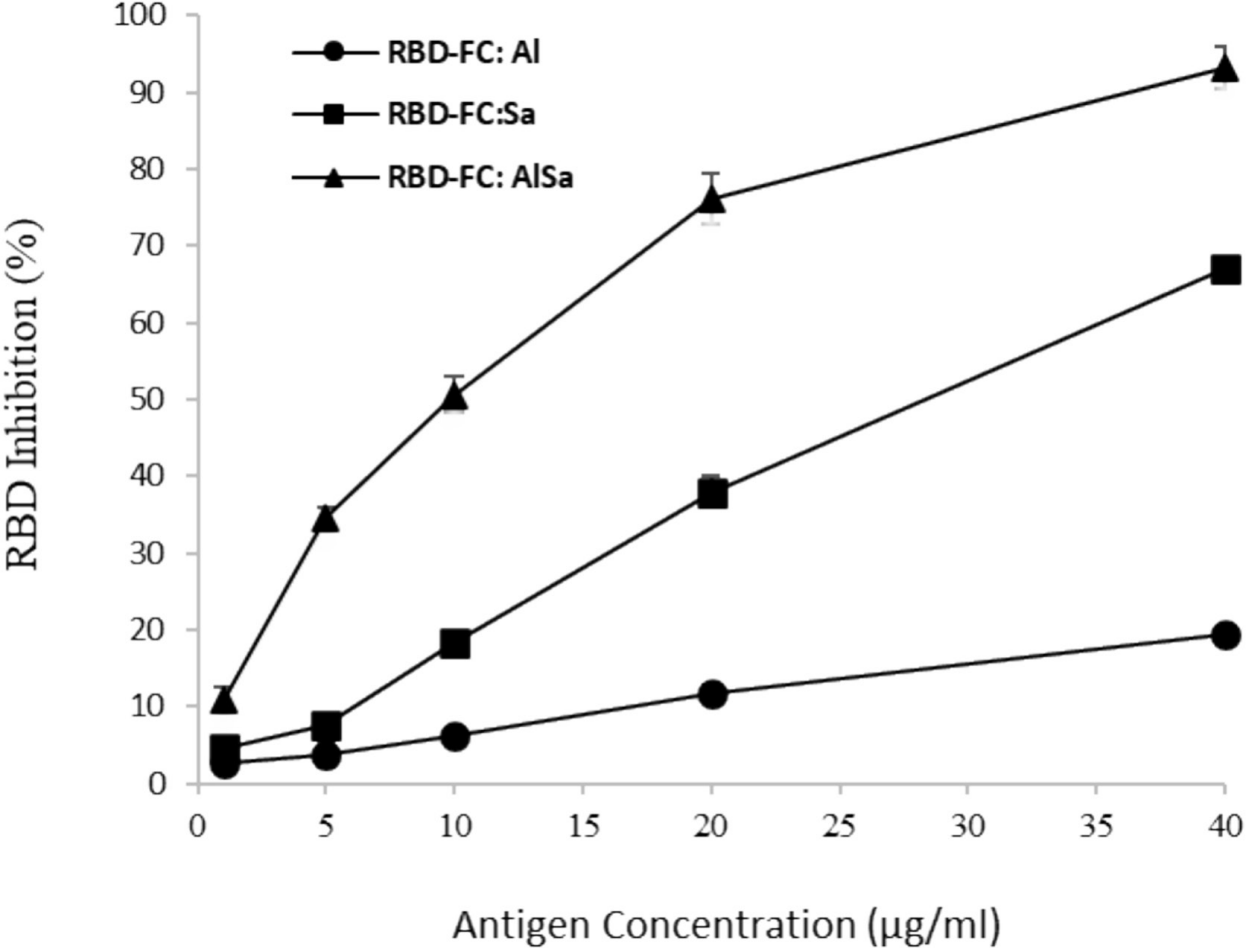
Inhibition ELISA showed combination of alum and sodium alginate plays a major role in production of anti‐RBD in mice sera.

The specificity of the antibody titer induced in the serum of immunized mice was evaluated by inhibition ELISA. In this method, a comparative analysis was carried out among anti‐RBD of mice sera after immunization with free RBD in different concentration against fixed RBD. In the highest antigen concentration (40 μg/mL), immunized mice with RBD:AlSa, RBD:Sa, and RBD:Al formulations demonstrated inhibitions of 93.1%, 67.2%, and 19.4%, respectively (Figure 5). Based on that, RBD:AlSa showed the highest ability to produced anti‐RBD agent than the other adjuvants. As depicted in Figure 5, combination of alum and sodium alginate successfully increased anti‐RBD in mice sera. Our presented data suggested that there is a close relationship between the induced antibody and recombinant RBD.

### 3.5 SARS‐CoV‐2 sVNT

**FIGURE 6:**
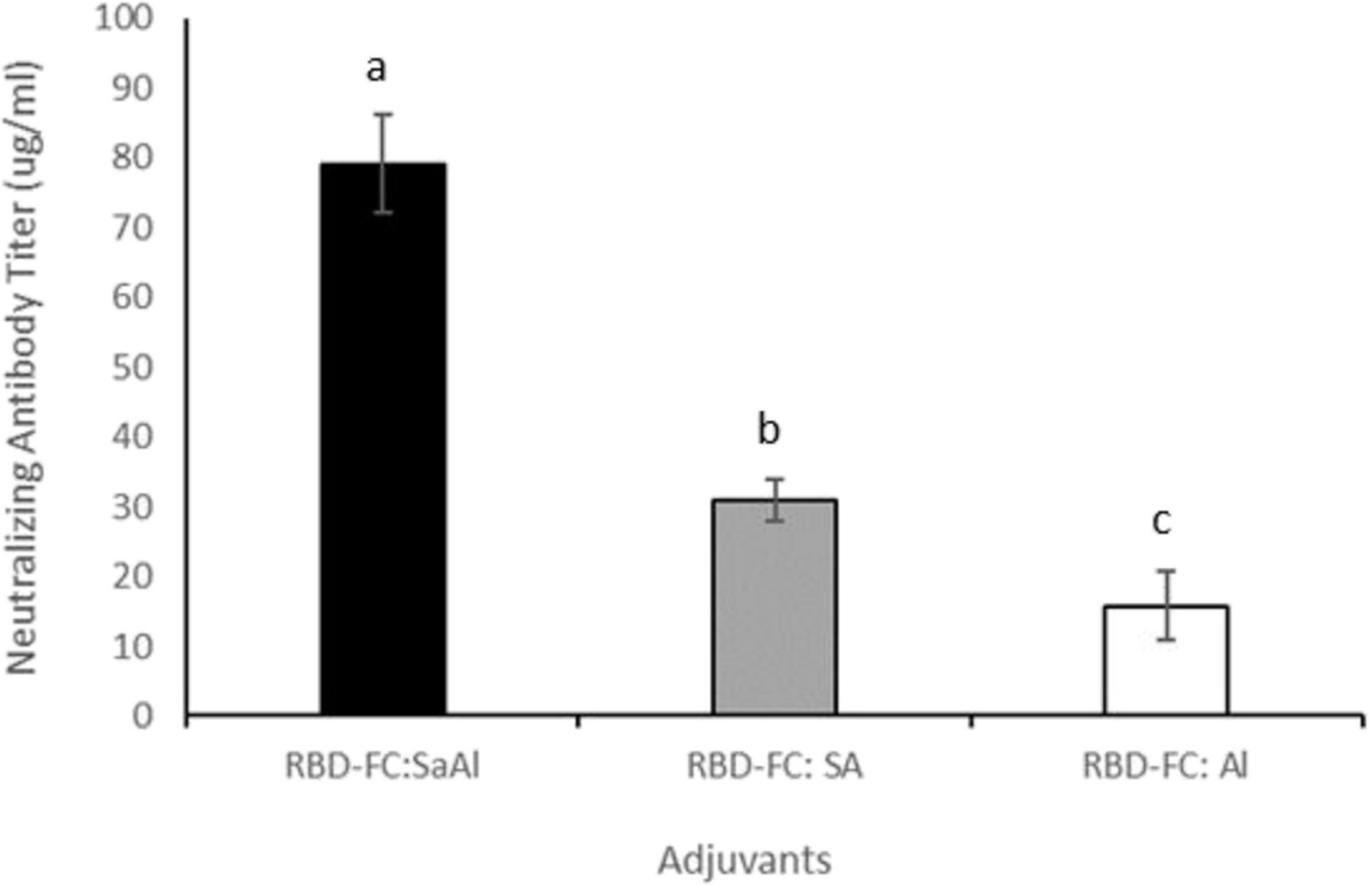
Neutralizing antibody titer of immunized mice sera with RBD:Al, RBD:Sa, and RBD:AlSa. As shown, AlSa significantly produced neutralizing antibody titer in mice.

As we know, sVNT is a powerful tool to detect neutralizing antibodies where conventional neutralization test (cVNTs) is lacking. According to the sVNT kit, the amount of neutralizing antibody greater than 2.5 μg/mL is considered as positive. Our data showed all three vaccine formulations (RBD:AlSa, RBD:Sa, and RBD:Al) generated the neutralizing antibody value more than 2.5 μg/mL. RBD:AlSa was able to generate neutralizing antibody up to 79.2 μg/mL, which was more than two and four times in comparison with RBD:Sa and RBD:Al, respectively (Figure 6).

### 3.6 Cytokine Assessment

**FIGURE 7:**
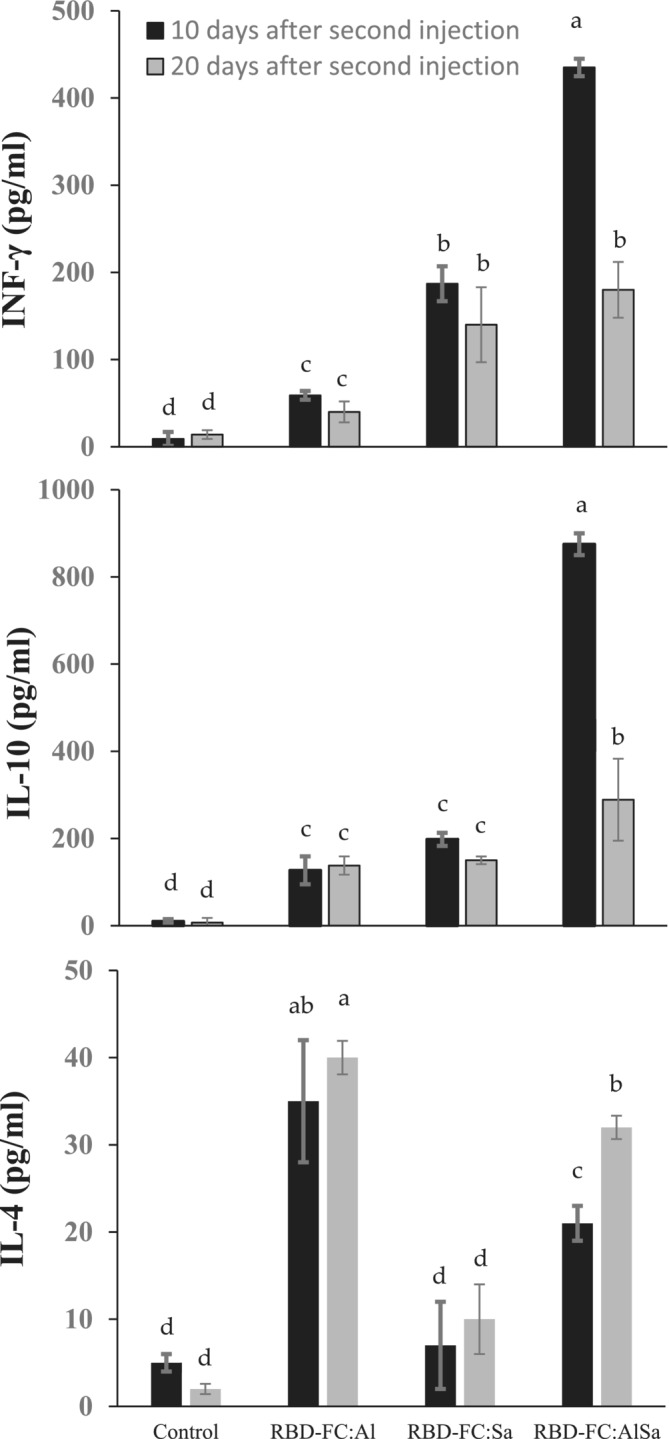
Effect of different vaccine formulations on the cytokine concentration of mouse serum at the two sampling times (10 and 20 days after second injection). As shown, adjuvant AlSa was the most effective to severe induction of cytokines INF‐γ and IL‐10 at 10 days after second injection, and after 20 days, amount of the cytokines significantly decreased. As seen, alum interestingly increased effectiveness of sodium alginate. Cytokine IL‐4 showed the lowest amount (pg/mL) among cytokines, and alginate had the lowest induction effect on IL‐4.

Cytokines play a major role in regulation of cell communications [36]. Cytokine activity determines intrinsic inflammation and anti‐inflammation status. Among cytokine players, IL‐4, IL‐10, and INF‐γ were assessed to characterized mouse sera response to different vaccine formulations at Days 10 and 20 after second injection. Under control condition (saline injection), all cytokines were at the lowest concentration level (Figure 7). Vaccination with RBD‐AlSa significantly increased the serum cytokine levels 10 days after second injection so that concentration of IL‐10 and INF‐γ reached to 875 and 435 pg/mL, respectively. In spite of IL‐10 and INF‐γ, IL‐4 content was at the highest level in response to RBD‐Al formulation. Sodium alginate did not have any significant effects on IL‐4 in comparison with control injection. Twenty days after second injection showed a significant effect from AlSa adjuvant on the all‐measured cytokines in comparison with 10 days after second injection. In a general view, combination of alum with sodium alginate made the most effective vaccine formulation for the highest accumulation of anti‐inflammatory cytokines in mouse serum at Day 10 after second injection.

## 4 Discussion

The present study was conducted to develop stable formulation of RBD‐FC recombinant protein against SARS‐CoV‐2. We employed *P. pastoris* for optimal and reliable synthesis of RBD‐FC protein. Expression of secretory RBD in *P. pastoris* is considered as effective approach. In Darvish et al. [30], who expressed RBD in *Escherichia coli* , comparative analysis of two expression platforms, *E. coli* BL21 and yeast KM71H strains, showed that *P. pastoris* with the maximal yield of RBD production (46 mg/L) and purity near to 95% was a good alternative for the expression of the highly glycosylated viral proteins [37].

An adjuvant is defined as a key component in vaccine formulation capable of enhancing and/or shaping antigen‐specific immune responses [38]. Alum and sodium alginate are the most famous adjuvants used in vaccine formulation for decades [39]. As we examined, the immunogenicity of RBD protein was significantly affected by vaccine adjuvants [30]. Sodium alginate significantly inhibited decrease of loading efficacy, whereas alum declined in loading efficacy of up to 25% during 6 months (Figure 3). It seems that alginate properties improved interaction of RBD‐FC with alginate hydrogels, but addition of alum to sodium alginate (AlSa) did not create a significant impact on the loading efficacy during 6 months. Our data showed that adjuvants AlSa and Sa had more loading efficacy than what Darvish et al. report [30]. It may be suggested that expression of the glycoprotein RBD‐FC in eukaryotic platform with glycosylation ability exerts a positive impact on the loading efficacy results. Alginate and alum interaction caused a significant elicitation in anti‐RBD IgG, so alginate alone up to twofold and alginate and alum together up to eightfold made an increase in antibody titer in comparison with alum adjuvant alone (Figure 4). It seems that alum could improve gel formation of sodium alginate and consequently increased elicitation of anti‐RBD IgG in mice sera. The same result was reported in combination of calcium and sodium alginate [21]. As Darvish et al. previously reported [30], combination of aluminum hydroxide and sodium alginate improved the immunogenicity of RBD vaccine produced in *E. coli* platform but our data suggested that interaction of AlSa with RBD‐FC derived from yeast expression system is more efficient in induction of immunogenicity. As it was expected, inhibition assay (Figure 5) and SARS‐CoV‐2 surrogate virus neutralization test (Figure 6) demonstrated excellent performance of AlSa adjuvant. This result was the same as Darvish et al. [30] report.

Cytokines generally reflect inflammation status of the body tissues. In 2021, Liu et al. comprehensively studied intercorrelated cytokine network among different groups of patients with COVID‐19 [40]. They classified cytokines in different groups, and IL‐10 and INF‐γ showed a maximum accumulation, and IL‐4 had a minimum accumulation in the deceased patients in three distinct groups. With this basis, we analyzed the response of immunized mice by differential accumulation of the three cytokines as candidates of cytokine network. As shown in Figure 7, AlSa adjuvant generated the highest changes in IL‐10 and INF‐γ, and after that, alginate and alum had the significant effects on the content of these cytokines, respectively. In response to AlSa, amount of the two cytokines significantly declined at Day 20 after second injection. Ten days after second injection, severe accumulation of IL‐4 was measured, and its accumulation was stable until 20 days after second injection. AlSa significantly increased IL‐4 concentration at 10 and 20 days after second injections. When we compared cytokine response between our RBD‐FC vaccine expressed in *P. pastoris* and vaccines presented by Darvish et al. [30] and Abdelallah et al. [19], accumulation amount of IL‐10 and INF‐γ in our formulation was more than them, and it seems that RBD‐FC conjugation with AlSa adjuvant is more efficient than others. As discussed in Lima et al. [41], approved adjuvants are able to increase immunogenicity of SARS‐CoV‐2 RBD, which might be defendable that the glycosylated RBD‐FC vaccine formulated with AlSa adjuvant is the best to increase immunogenicity and modulating immune response against SARS‐CoV‐2.

## Author Contributions

Mahboobeh Dehghan carried out all experiments and all data collection and prepared first version of manuscript. Masoud Tohidfar made data analysis/interpretation. Seyed Omid Ranaei Siadat carried out the conception/design of the research. Hossein Askari carried out drafting the manuscript and making intellectual contributions on text/revisions and final approval of the manuscript. Fataneh Fatemi carried out primary design of the research.

## Ethics Statement

Animal research were approved by Iran National Committee by Ethics in Biomedical Research.

## Consent

No patient data are involved in this article.

## Conflicts of Interest

The authors declare no conflicts of interest.

### Peer Review

The peer review history for this article is available at https://www.webofscience.com/api/gateway/wos/peer‐review/10.1111/irv.70018.

## Data Availability

All data supporting the findings of this study are available within the article.

## References

[1] A. Zumla , J. F. Chan , E. I. Azhar , D. S. Hui , and K.‐Y. Yuen , “Coronaviruses—Drug Discovery and Therapeutic Options,” Nature Reviews Drug Discovery 15, no. 5 (2016): 327–347. 26868298 10.1038/nrd.2015.37 PMC7097181

[2] P. Zhou , X.‐L. Yang , X.‐G. Wang , et al., “A Pneumonia Outbreak Associated With a New Coronavirus of Probable Bat Origin,” Nature 579, no. 7798 (2020): 270–273. 32015507 10.1038/s41586-020-2012-7 PMC7095418

[3] D. Sanyal , S. Chowdhury , V. N. Uversky , and K. Chattopadhyay , “An Exploration of the SARS‐CoV‐2 Spike Receptor Vinding Domain (RBD)–A Complex Palette of Evolutionary and Structural Features,” BioRxiv, (2020), 2020.05. 31.126615. 10.1016/j.ijbiomac.2022.07.022 PMC9278002 35841961

[4] S. Xia , M. Liu , C. Wang , et al., “Inhibition of SARS‐CoV‐2 (Previously 2019‐nCoV) Infection by a Highly Potent pan‐Coronavirus Fusion Inhibitor Targeting Its Spike Protein That Harbors a High Capacity to Mediate Membrane Fusion,” Cell Research 30, no. 4 (2020): 343–355. 32231345 10.1038/s41422-020-0305-x PMC7104723

[5] N. Wang , J. Shang , S. Jiang , and L. Du , “Subunit Vaccines Against Emerging Pathogenic Human Coronaviruses,” Frontiers in Microbiology 11 (2020): 298. 32265848 10.3389/fmicb.2020.00298 PMC7105881

[6] Y. He , Y. Zhou , S. Liu , et al., “Receptor‐Binding Domain of SARS‐CoV Spike Protein Induces Highly Potent Neutralizing Antibodies: Implication for Developing Subunit Vaccine,” Biochemical and Biophysical Research Communications 324, no. 2 (2004): 773–781. 15474494 10.1016/j.bbrc.2004.09.106 PMC7092904

[7] Y. He , J. Li , W. Li , S. Lustigman , M. Farzan , and S. Jiang , “Cross‐Neutralization of Human and Palm Civet Severe Acute Respiratory Syndrome Coronaviruses by Antibodies Targeting the Receptor‐Binding Domain of Spike Protein,” The Journal of Immunology 176, no. 10 (2006): 6085–6092. 16670317 10.4049/jimmunol.176.10.6085

[8] Y. Kato , H. Onishi , and Y. Machida , “Application of Chitin and Chitosan Derivatives in the Pharmaceutical Field,” Current Pharmaceutical Biotechnology 4, no. 5 (2003): 303–309. 14529420 10.2174/1389201033489748

[9] L. Du , G. Zhao , Y. He , et al., “Receptor‐Binding Domain of SARS‐CoV Spike Protein Induces Long‐Term Protective Immunity in an Animal Model,” Vaccine 25, no. 15 (2007): 2832–2838. 17092615 10.1016/j.vaccine.2006.10.031 PMC7115660

[10] Y. M. Vasiliev , “Chitosan‐Based Vaccine Adjuvants: Incomplete Characterization Complicates Preclinical and Clinical Evaluation,” Expert Review of Vaccines 14, no. 1 (2015): 37–53. 25262982 10.1586/14760584.2015.956729

[11] S. L. Leary , W. Underwood , R. Anthony , et al., eds., AVMA Guidelines for the Euthanasia of Animals, 2013 edition (Schaumburg, IL: American Veterinary Medical Association, 2013).

[12] O. S. Koutsoni , M. Barhoumi , I. Guizani , and E. Dotsika , “New Insights on the Adjuvant Properties of the *Leishmania infantum* Eukaryotic Initiation Factor,” Journal of Immunology Research 2019 (2019): 1–13. 10.1155/2019/9124326 PMC6515109 31183394

[13] N. J. Horscroft , D. C. Pryde , and H. Bright , “Antiviral Applications of Toll‐Like Receptor Agonists,” The Journal of Antimicrobial Chemotherapy 67, no. 4 (2012): 789–801. 22258929 10.1093/jac/dkr588

[14] N. B. Sarikahya , A. Nalbantsoy , H. Top , R. S. Gokturk , H. Sumbul , and S. Kirmizigul , “Immunomodulatory, Hemolytic and Cytotoxic Activity Potentials of Triterpenoid Saponins From Eight *Cephalaria* Species,” Phytomedicine 38 (2018): 135–144. 29425646 10.1016/j.phymed.2017.11.009

[15] S. Mallakpour , E. Azadi , and C. M. Hussain , “Chitosan, Alginate, Hyaluronic Acid, Gums, and β‐Glucan as Potent Adjuvants and Vaccine Delivery Systems for Viral Threats Including SARS‐CoV‐2: A Review,” International Journal of Biological Macromolecules 182 (2021): 1931–1940. 34048834 10.1016/j.ijbiomac.2021.05.155 PMC8146404

[16] D. Christensen , C. Foged , I. Rosenkrands , H. M. Nielsen , P. Andersen , and E. M. Agger , “Trehalose Preserves DDA/TDB Liposomes and Their Adjuvant Effect During Freeze‐Drying,” Biochimica et Biophysica Acta (BBA) ‐ Biomembranes 1768, no. 9 (2007): 2120–2129. 17555704 10.1016/j.bbamem.2007.05.009

[17] F. Coumes , C.‐Y. Huang , C.‐H. Huang , et al., “Design and Development of Immunomodulatory Antigen Delivery Systems Based on Peptide/PEG–PLA Conjugate for Tuning Immunity,” Biomacromolecules 16, no. 11 (2015): 3666–3673. 26473322 10.1021/acs.biomac.5b01150

[18] F. Dobakhti , T. Naghibi , M. Taghikhani , et al., “Adjuvanticity Effect of Sodium Alginate on Subcutaneously Injected BCG in BALB/c Mice,” Microbes and Infection 11, no. 2 (2009): 296–301. 19110068 10.1016/j.micinf.2008.12.003

[19] N. H. AbdelAllah , N. F. Abdeltawab , A. A. Boseila , and M. A. Amin , “Chitosan and Sodium Alginate Combinations Are Alternative, Efficient, and Safe Natural Adjuvant Systems for Hepatitis B Vaccine in Mouse Model,” Evidence‐Based Complementary and Alternative Medicine 2016, no. 1 (2016): 7659684. 27493674 10.1155/2016/7659684 PMC4963576

[20] A. Itodo , J. Umoh , J. Adekeye , M. Odugbo , G. Haruna , and M. Sugun , “Field Trial of Sodium Alginate‐Adsorbed *Clostridium perfringens* Types C and D Toxoid Against Clostridial Enterotoxemia in Sheep,” Israel Journal of Veterinary Medicine 64, no. 1 (2009): 2.

[21] G. H. Scherr , A. S. Markowitz , and L. Skelton , “A New Alginate Adjuvant,” Journal of Applied Microbiology 28, no. 1 (1965): 174–180.

[22] A. Shapiro , Y. Modai , and A. Kohn , “Efficacies of Vaccines Containing Alginate Adjuvant,” Journal of Applied Microbiology 30, no. 2 (1967): 304–311. 10.1111/j.1365-2672.1967.tb00301.x 6073987

[23] B. Ray , I. Ali , S. Jana , et al., “Antiviral Strategies Using Natural Source‐Derived Sulfated Polysaccharides in the Light of the COVID‐19 Pandemic and Major Human Pathogenic Viruses,” Viruses 14, no. 1 (2021): 35. 35062238 10.3390/v14010035 PMC8781365

[24] A. Cano‐Vicent , R. Hashimoto , K. Takayama , and Á. Serrano‐Aroca , “Biocompatible Films of Calcium Alginate Inactivate Enveloped Viruses Such as SARS‐CoV‐2,” Polymers 14, no. 7 (2022): 1483. 35406356 10.3390/polym14071483 PMC9002394

[25] D. Slavin , “Production of Antisera in Rabbits Using Calcium Alginate as an Antigen Depot,” Nature 165, no. 4186 (1950): 115–116. 15404044 10.1038/165115a0

[26] C. Amies , “The Use of Topically Formed Calcium Alginate as a Depot Substance in Active Immunisation,” Journal of Pathology and Bacteriology 77 (1959): 435–441. 13642191 10.1002/path.1700770214

[27] H. H. Flowers , “Active Immunization of a Human Being Against Cobra (*Naja naja*) Venom,” Nature 200, no. 4910 (1963): 1017–1018. 10.1038/2001017b0 14097729

[28] S. Jaros and J. Dewey , “Uses of an Alginate in Hyposensitization,” Annals of Allergy 22 (1964): 173–179. 14142447

[29] Á. Serrano‐Aroca , M. Ferrandis‐Montesinos , and R. Wang , “Antiviral Properties of Alginate‐Based Biomaterials: Promising Antiviral Agents Against SARS‐CoV‐2,” ACS Applied Bio Materials 4, no. 8 (2021): 5897–5907. 10.1021/acsabm.1c00523 35006918

[30] M. Darvish , Z. Moosavi‐Nejad , S. O. R. Siadat , F. Fatemi , and A. Khatibi , “Enhancing Neutralizing Antibodies Against Receptor Binding Domain of SARS‐CoV‐2 by a Safe Natural Adjuvant System,” Virus Research 326 (2023): 199047. 36693449 10.1016/j.virusres.2023.199047 PMC9867563

[31] R. Shirvani , S. Yazdanpanah , M. Barshan‐Tashnizi , and M. Shahali , “A Novel Methanol‐Free Platform for Extracellular Expression of rhGM‐CSF in *Pichia pastoris* ,” Molecular Biotechnology 61 (2019): 521–527. 31054084 10.1007/s12033-019-00182-6

[32] J. Zang , Y. Zhu , Y. Zhou , et al., “Yeast‐Produced RBD‐Based Recombinant Protein Vaccines Elicit Broadly Neutralizing Antibodies and Durable Protective Immunity Against SARS‐CoV‐2 Infection,” Cell Discovery 7, no. 1 (2021): 71. 34408130 10.1038/s41421-021-00315-9 PMC8372230

[33] W. Zhang , B. Y. Chua , K. J. Selva , et al., “SARS‐CoV‐2 Infection Results in Immune Responses in the Respiratory Tract and Peripheral Blood That Suggest Mechanisms of Disease Severity,” Nature Communications 13, no. 1 (2022): 2774. 10.1038/s41467-022-30088-y PMC9120039 35589689

[34] O. Borges , M. Silva , A. de Sousa , G. Borchard , H. E. Junginger , and A. Cordeiro‐da‐Silva , “Alginate Coated Chitosan Nanoparticles Are an Effective Subcutaneous Adjuvant for Hepatitis B Surface Antigen,” International Immunopharmacology 8, no. 13–14 (2008): 1773–1780. 18801462 10.1016/j.intimp.2008.08.013

[35] A. Raghav , J. Ahmad , and K. Alam , “Nonenzymatic Glycosylation of Human Serum Albumin and Its Effect on Antibodies Profile in Patients With Diabetes Mellitus,” PLoS ONE 12, no. 5 (2017): e0176970. 28520799 10.1371/journal.pone.0176970 PMC5435419

[36] J.‐M. Zhang and J. An , “Cytokines, Inflammation and Pain,” International Anesthesiology Clinics 45, no. 2 (2007): 27–37. 17426506 10.1097/AIA.0b013e318034194e PMC2785020

[37] C.‐P. Chuck , C.‐H. Wong , L. M.‐C. Chow , K.‐P. Fung , M. M.‐Y. Waye , and S. K.‐W. Tsui , “Expression of SARS‐Coronavirus Spike Glycoprotein in Pichia Pastoris,” Virus Genes 38 (2009): 1–9. 18958613 10.1007/s11262-008-0292-3 PMC7088578

[38] S. G. Reed , M. T. Orr , and C. B. Fox , “Key Roles of Adjuvants in Modern Vaccines,” Nature Medicine 19, no. 12 (2013): 1597–1608. 10.1038/nm.3409 24309663

[39] N. Petrovsky and J. C. Aguilar , “Vaccine Adjuvants: Current State and Future Trends,” Immunology and Cell Biology 82, no. 5 (2004): 488–496. 15479434 10.1111/j.0818-9641.2004.01272.x

[40] Y. Liu , D. Chen , J. Hou , et al., “An Inter‐correlated Cytokine Network Identified at the Center of Cytokine Storm Predicted COVID‐19 Prognosis,” Cytokine 138 (2021): 155365. 33246770 10.1016/j.cyto.2020.155365 PMC7651249

[41] G. G. Lima , A. I. Portilho , and E. De Gaspari , “Adjuvants to Increase Immunogenicity of SARS‐CoV‐2 RBD and Support Maternal–Fetal Transference of Antibodies in Mice,” Pathogens and Disease 80, no. 1 (2022): ftac038. 36220147 10.1093/femspd/ftac038 PMC9620730

